# Can Urine Metabolomics Be Helpful in Differentiating Neuropathic and Nociceptive Pain? A Proof-of-Concept Study

**DOI:** 10.1371/journal.pone.0150476

**Published:** 2016-03-02

**Authors:** Gabriele Finco, Emanuela Locci, Paolo Mura, Roberta Massa, Antonio Noto, Mario Musu, Giovanni Landoni, Ernesto d’Aloja, Fabio De-Giorgio, Paola Scano, Maurizio Evangelista

**Affiliations:** 1 Department of Medical Sciences “M. Aresu”, University of Cagliari, Cagliari, Italy; 2 Department of Public Health, Clinical and Molecular Medicine, University of Cagliari, Cagliari, Italy; 3 Department of Surgery, University of Cagliari, Cagliari, Italy; 4 Department of Anesthesia and Intensive Care, Vita-Salute San Raffaele University, Milan, Italy; 5 Public Health Institute, Catholic University of Rome, Rome, Italy; 6 Department of Chemical and Geological Sciences, University of Cagliari, Cagliari, Italy; 7 Department of Emergency, Catholic University of Rome, Rome, Italy; Mayo Clinic, UNITED STATES

## Abstract

The diagnosis of pain nature is a troublesome task and a wrong attribution often leads to an increase of costs and to avoidable pharmaceutical adverse reactions. An objective and specific approach to achieve this diagnosis is highly desirable. The aim of this work was to investigate urine samples collected from patients suffering from pain of different nature by a metabolomics approach based on ^1^H NMR spectroscopy and multivariate statistical analysis. We performed a prospective study on 74 subjects: 37 suffering from pain (12 with nociceptive and 25 with neuropathic pain), and 37 controls not suffering from any kind of chronic pain. The application of discriminant analysis on the urine spectral profiles allowed us to classify these two types of pain with high sensibility and specificity. Although the classification relies on the global urine metabolic profile, the individual contribution in discriminating neuropathic pain patients of metabolites such as choline and phosphocholine, taurine and alanine, suggests potential lesions to the nervous system. To the best of our knowledge, this is the first time that a urine metabolomics profile is used to classify these two kinds of pain. This methodology, although based on a limited sample, may constitute the basis for a new helpful tool in the clinical diagnosis.

## Introduction

The diagnosis of chronic pain is still challenging for physicians in the everyday clinical setting. This difficulty is due to the absence of a universal agreement about the description and classification of the different typologies of pain [1]. The International Association for the Study of Pain (IASP) defines pain as an individual, sensorial and subjective experience, characterized by both mental and physical factors, combined with a variety of other symptoms [2]. It may be difficult to distinguish nociceptive pain, resulting from tissue damage (mostly inflammatory in nature), and neuropathic pain, involving nervous system damage. There is no gold standard for this issue [3], and often patients receive a diagnosis of mixed or uncertain pain. The recommended approach to pain diagnosis relies on a “stepwise process” that includes an accurate anamnesis, an in depth neurological examination, the performance of appropriate diagnostic tests, and the application of pain questionnaires [2,4–8], but a certain diagnosis is reached in only about 80% of cases [1,9]. If the diagnostic process leads to a wrong result, the treatment will be unsatisfactory and treating the patient with an inappropriate therapy will increase the direct and indirect costs of the illness and side effects will be unjustified.

The gold standard, as above defined, is unsatisfactory in differentiating pain types, especially for neuropathic pain [1]. Many scientists are looking for clinical features, laboratory markers or instrumental signs characteristic of this kind of chronic pain and uninfluenced by the subjectivity of either the patient or the physician. So far, their efforts have failed to produce convincing and unambiguous results that can be translated into clinical practice [10].

Among the emerging technologies, metabolomics may be a useful tool to identify a biological signature that discriminates among different pain syndromes, helping in the diagnosis and treatment of patients. Metabolomics is defined as the study of the complete set of low molecular weight metabolites (metabolome) within a biological fluid [11]. High Resolution ^1^H Nuclear Magnetic Resonance (NMR) spectroscopy is very attractive since it is highly reproducible and requires minimal sample preparation. Recent evidences, based on metabolomics with different analytical platforms (^1^H NMR, Mass Spectrometry), on different biological matrices (plasma, urine, cerebrospinal fluid, and tissues), from either humans or animal models [12–18], suggested that this approach could be a promising tool to help in the diagnostic assessment of pain. To the best of our knowledge, difference between neuropathic and nociceptive pain has never been investigated. Therefore, the objective of our study was to assess whether urine metabolomics profiles can differentiate neuropathic and nociceptive pain, as diagnosed on the basis of the current clinical protocol. To this goal, we applied a metabolomics approach based on ^1^H NMR analysis of urine collected from patients affected by these two different types of pain and from a control group.

## Materials and Methods

### Patients and controls

This prospective study was conducted in December 2014 on consecutive patients referred to the Pain Therapy Centres of University Hospital of Cagliari and of Columbus Clinic of the Catholic University of Rome, both in Italy. Approval by the Institutional Review Board (Comitato Etico Indipendente dell’Azienda Ospedaliero-Universitaria di Cagliari, November 17^th^, 2014) was obtained. Written informed consent was obtained from all subjects. Inclusion criteria were all of the following: presence of pain lasting for more than 3 months; main pain intensity in the last month ≥ 3 on a 0–10 Number Rating Scale (NRS); age ≥ 18 years; written informed consent; first visit at the Pain Centres. The exclusion criteria were at least one of the following: cancer related pain; psychiatric disorders; participation in clinical trials in the last 30 days. Ambulatory nurse administered the PainDETECT Questionnaire (PDQ) and two experienced pain specialists independently examined all the patients in accordance with the current gold standard [6] to discriminate the pain as predominantly nociceptive or neuropathic. In case of different opinion between the two specialists, PDQ results were used to settle the conflict. Control urine samples were randomly chosen, from patients willing to contribute to the research. Those patients presented and required clinical analysis in our hospital, and were not suffering from any kind of chronic pain, neither neuropathic nor nociceptive. They were mostly surgical patients scheduled for elective endocrine and gynaecological surgery (mostly thyroidectomy and hysterectomy). Unfortunately, the epidemiological and demographic characteristics of this kind of patients, prevented us to perfectly match for gender and age our control sample with pain patients. Then, for each model, the best-balanced age matching available between cases and controls has been performed, considering for age the slightest difference available.

### Sample collection and preparation for ^1^H NMR spectroscopy

At the moment of urine collection patients were fasting for at least 6 hours. Centrifugation of samples at 13.000 rpm for 10 minutes at 4°C was followed by supernatants collection and 10 μL of a 10% w/w aqueous solution of sodium azide (NaN_3_) were added to 1 mL of supernatant. Samples were immediately frozen at -80°C until NMR analysis. For ^1^H NMR analysis, 1 mL of thawed urine was centrifuged again. An aliquot of 630 μl was mixed to 70ul of a 1.5 M phosphate buffer solution (pH = 7.4) in D_2_O (99.9%, Cambridge Isotope Laboratories Inc, Andover, USA) containing the internal standard sodium 3-(trimethylsilyl)propionate-2,2,3,3,-*d*_*4*_ (TSP, 98 atom % D, Sigma-Aldrich, Milan) at a 0.6 mM final concentration, and 650 μl of the obtained solution were transferred into a 5 mm NMR tube.

### ^1^H NMR data acquisition and processing

^1^H NMR experiments were carried out on a Varian UNITY INOVA 500 spectrometer (Agilent Technologies, CA, USA). Spectra were recorded using a 1D-NOESY pulse sequence with a mixing time of 1 ms and a recycle time of 3.5 s, for water suppression. Spectra were acquired at 300K, with a spectral width of 6000 Hz, a 90° pulse, and 128 scans. Before Fourier transformation, the free induction decays (FID) were zero-filled to 64k and an exponential weighting function was applied with a line-broadening factor of 0.5 Hz. Spectra were manually phased and baseline corrected using MestReNova software (Version 9.0, Mestrelab Research S.L.). Chemical shifts were referred to the TSP single resonance at 0.00 ppm. The assignment of major resonances was performed on the basis of data published in the literature [19] and using the database implemented in Chenomx NMR suite 7.1 (Chenomx Inc., Edmonton, Alberta, Canada). The ^1^H NMR spectra were reduced to consecutive integrated spectral regions (bins) of equal width (0.04 ppm) corresponding to the region 0.5–9.5 ppm. The spectral region between 4.50 and 6.50 ppm was excluded from the analysis to remove artifacts arising from water signal suppression, the broad urea resonance and spectral noise. The binning procedure was performed by MestReNova. To minimize the effects of variable concentration among different samples, for each spectrum the bin integrated areas were normalized to 100. The final data matrix was imported into the SIMCA-P+ (Version 13.0, Umetrics, Umeå, Sweden) statistical software package.

### Statistical analysis

#### Multivariate statistical analysis of ^1^H NMR spectral data

Multivariate statistical analysis of ^1^H NMR spectral data were performed by the unsupervised Principal components Analysis (PCA) and the supervised Partial Least Square Discriminant Analysis (PLS-DA) and its Orthogonal variant (OPLS-DA), as implemented in SIMCA software package. PCA is an explorative chemometric tool useful to check trends between samples and variables, presence of outliers, and deviating features in the data. PLS-DA is a classification tool based on PLS approach where the **y**-vector is the class label, in pairwise DA the dummy variables, 0 and 1, are given to the two classes. OPLS-DA is a variant of PLS-DA, where separation between classes is forced in the first component (the “predictive” component) and further components, orthogonal to the first, only express intra-class variability. The classification power of models is expressed by the correlation coefficient R^2^Y. The DA models were validated by performing a series of tests such as cross-validation, in which samples are split into groups (taking care that in each set, samples of both classes were present) and their member-ship predicted, result of this procedure is the parameter Q^2^Y (the classification power in cross-validation). A Q^2^Y value very different from R^2^Y is index of an overfitting in the original model, a difference value larger than 0.3 has to be taken with caution. Results of the permutation test with n = 400 were also checked [20]. The appropriate number of PCs for each model was based on cross validation, in fact, although the variance explained by the model (R^2^X and R^2^Y, for PCA and PLS-DA, respectively) will increase with subsequent components, the classification power of the model in cross-validation (Q^2^) does not automatically increase with incremental components, and thereafter, the inclusion of subsequent components into the model will only add noise. Results of models can be shown as score plot where samples are projected in the multivariate space, while influence of a variable in the classification can be inferred by its magnitude (modelled covariation) and reliability (modelled correlation) values for a predetermined class. High reliability means high effect and lower uncertainty for the variable (putative biomarker), indeed, taking into account model correlations, it is possible to investigate the reliability of small signals, that can contain precious information, not highlighted by the model covariance under Pareto scaling [21].

#### Contingency table

PLS-DA is a hard modelling tool in which to each object/sample an estimate of class membership to only one of the specified classes is given. Results of pairwise OPLS-DA models have, therefore, binary outcome. As implemented in SIMCA software, a value of prediction of belonging to one of the predefined classes is given for each sample. A value close to one and above 0.65 indicate membership of the sample to the predefined class, values between 0.35 and 0.65 that membership is borderline or uncertain, and values < 0.35 indicate that the sample does not belong to the class. These values were imported in a contingency table, using the clinical evaluation as gold standard to define classes. Using values > 0.65 and considering as wrong attribution borderline values (between 0.35 and 0.65), which is the most conservative way to discriminate membership, sensibility (true positive/affected), specificity (true negative/not affected), positive and negative predictive values (true positive/all positive, true negative/all negative, PPV and NPV, respectively) and accuracy (true positive and negative/total population) of the models [22], were calculated. Analysis were performed with InStat Prism 6.01, GraphPad^®^ Software, San Diego, California; USA. Statistical significance was determined by two tailed Fischer Exact Test, and a *p* < 0.05 was deemed as significant.

## Results

Urine samples were collected from patients diagnosed as suffering from nociceptive pain (NC, n = 12) and neuropathic pain (NP, n = 25) and from controls (C, n = 37). Demographic and clinical characteristics of pain patients and controls are reported in Table 1: women suffering pain outnumbered men and the mean age was higher in patients with nociceptive pain.

**Table 1:** Demographic and clinical characteristics of subjects.

	Sample size	Pain duration (months)^c^	PD score^d^	Gender (M/W)	Age (years)^d^
**Pain group**					
**NP**^a^	25	25 (18–30)	22.31 ± 7.09	17/8	65 ± 15
**NC**^b^	12	19 (8–25)	2.90 ± 2.23	9/3	71 ± 14
**Control group**	37			26/11	55 ± 13

NP, neuropathic; NC, nociceptive; M/W, men/women.

^a^Trigeminal Neuralgia (n = 2), Thalamic Syndrome (n = 1), Phantom Limb (n = 2), Spinal Stenosis (n = 2), Postherpetic Neuralgia (n = 4), Fibromyalgia (n = 1), Failed Low Back Surgery (n = 3), Diabetic Polyneuropathy (n = 1), CPRS I (n = 2), Postsurgical Pain (n = 4), Post Actinic Neuralgia (n = 1), Radicular Pain (n = 3).

^b^Low Back Pain (n = 2), Sacroileitis (n = 1), Polyarthritis (n = 6), Psoriasic Arthritis (n = 1), Gonalgie (n = 1), Coxarthritis (n = 1).

^c^values expressed as the median (range).

^d^values are expressed as the mean ± standard deviation.

**Fig 1:**
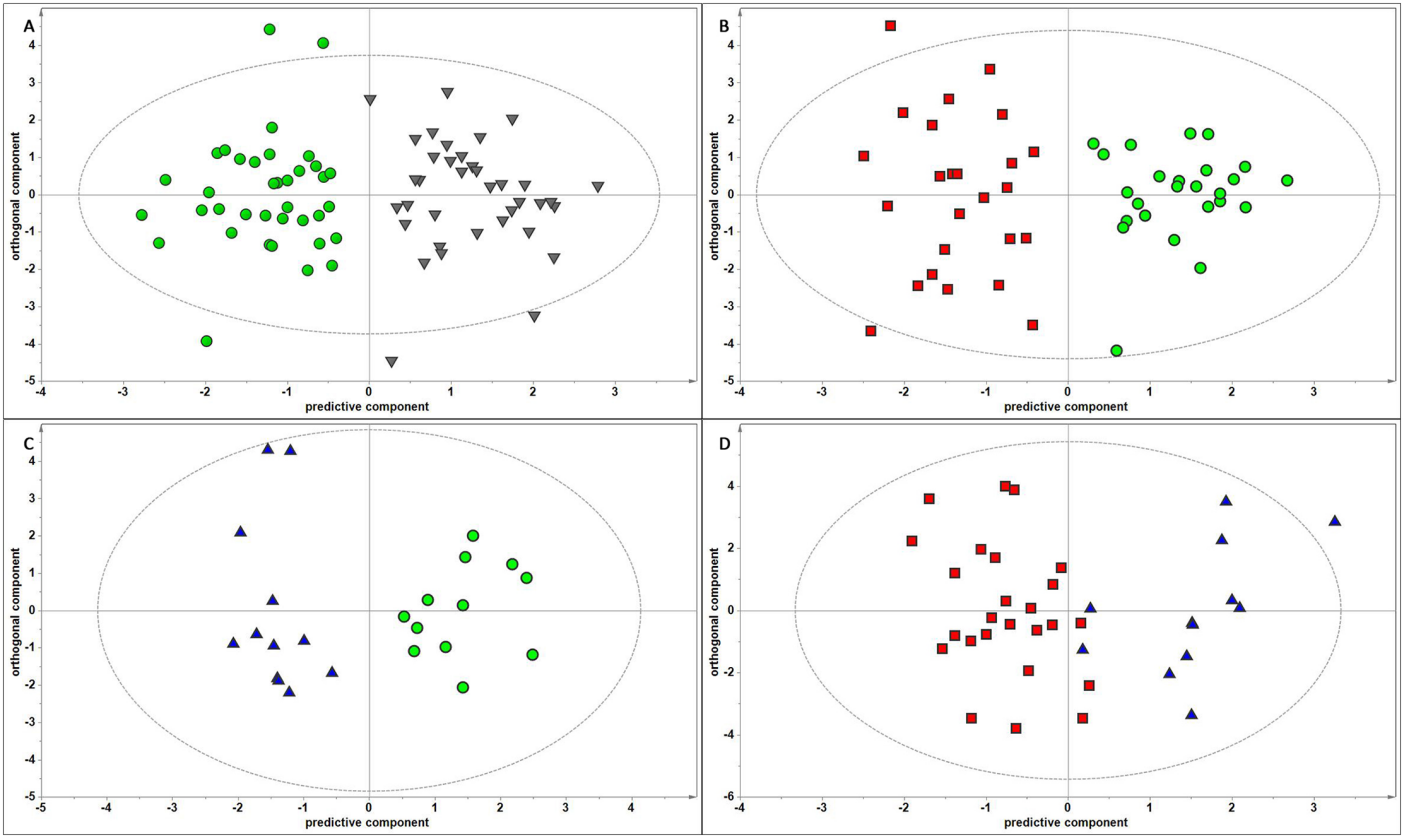
OPLS-DA score plots in the predictive (*x*-axis) and orthogonal (*y*-axis) components of ^1^H NMR spectral data of urine samples. **A) “Pain *vs*. C” model. B) “NP *vs*. C” model. C) “NC *vs*. C” model. D) “NP *vs*. NC” model.** Separation of classes is maximized along the predictive component, while the orthogonal component accounts for intra-class variability. Ellipse indicates the confidence region. Pain: neuropathic and nociceptive pain samples—inverted grey triangles; C: matched control samples—green circles; NP: neuropathic pain samples—red squares; NC nociceptive pain samples—blue triangles. Details of OPLS-DA models performance are given in S1 Table.

^1^H NMR spectra were recorded for all samples. A representative ^1^H NMR spectrum of a urine sample with major assignments is reported in S1 Fig. Spectral data were submitted to MVA. An initial overview of samples distribution was obtained by PCA. In the score plot of the first two (S2 Fig) components no grouping, no clusters, no trends of samples based on clinical diagnosis, or confounding factors as gender and age were observed. To assess the classification potential of the metabolomics approach, we carried out two-class DA and results of classification models in terms of value of prediction of belonging to one of the two predefined classes for each sample elaborated through the use of the contingency table. For each model, control samples were chosen with age and gender matching to those of individuals suffering of pain. At first, to ascertain whether subjects with pain syndromes have a different ^1^H NMR urine metabolite profile with respect to the random control population, we performed a two classes OPLS-DA, where both NP and NC samples were merged in an unique class: “pain”, and compared to C class. Among the variables characterizing the pain profile, we found also mannitol, which is a common excipient of several drugs currently employed in the treatment of pain syndromes. We therefore excluded the mannitol spectral region (from 3.62 to 3.90 ppm) from the data matrix and we performed a new OPLS-DA analysis. The resulting “Pain *vs*. C” model gave satisfactory results, in terms of good class separation and classification ability (the corresponding R^2^Y and Q^2^Y values are reported in Table 2) and the resulting score plot, that shows sample distribution in the multivariate space, is reported in Fig 1A.

**Table 2:** Results of samples classification for the different two-classes discriminant models.

	Number of Samples^a^			OPLS-DA parameters^b^		Contingency Table			
Model	NP	NC	C	R^2^Y	Q^2^Y	Sensitivity	Specificity	Accuracy	*p* value
**Pain vs. C**	25	12	37	0.80	0.66	0.86	0.95	0.90	<0.0001
**NP vs. C**	25	-	25	0.83	0.65	0.92	0.92	0.92	<0.0001
**NC vs. C**	-	12	12	0.88	0.64	0.67	0.92	0.79	0.01
**NP vs. NC**	25	12	-	0.74	0.41	0.88	0.83	0.86	<0.0001

^a^Neuropathic pain (NP), nociceptive pain (NC), matched controls (C).

^b^Classification power (R^2^Y), classification power in cross-validation (Q^2^Y) of models.

Through the use of the contingency table, applied to the values of prediction of a sample to belong to one of the two classes, sensitivity, specificity, accuracy and significance of the classification were calculated and results, reported in Table 2, were quite satisfactory (detailed contingency tables are reported in S2 Table). Based on these results, we can state that the ^1^H NMR metabolite profile can well classify subjects with pain syndromes from that of control population. Results of this model prompted us to find discriminant models able to classify the two typologies of pain. Two-classes OPLS-DA of ^1^H NMR metabolite profiles of subjects diagnosed as suffering of neuropathic pain were compared to the controls, matched for age and gender. R^2^Y and Q^2^Y of the validated OPLS-DA “NP *vs*. C” model, and the calculated sensitivity, specificity and accuracy are reported in Table 2. Analogously, pairwise OPLS-DA of ^1^H NMR metabolite profiles of subjects diagnosed as suffering of nociceptive pain were compared to the controls matched for age and gender. R^2^Y and Q^2^Y of the validated OPLS-DA “NC *vs*. C” model and the calculated sensitivity, specificity and accuracy are reported in Table 2. The score plots of these latter two models are shown in Fig 1B and 1C, respectively. At this point, we wanted to take on the challenging task of discriminating subjects diagnosed as suffering of neuropathic pain from those suffering of nociceptive pain. ^1^H NMR metabolite profile of NP and NC samples were compared. The OPLS-DA gave good performance, R^2^Y and Q^2^Y of the validated OPLS-DA “NP *vs*. NC” model and the calculated sensitivity, specificity and accuracy are reported in Table 2. The resulting score plot is shown in Fig 1D.

Although aimed at finding a good classification tool, we turned our attention also to the metabolites that mostly contribute to class discrimination. To this goal, we analyzed the variable loadings values along the predictive component of the OPLS-DA “NP *vs*. NC” model and we found that urine of patients classified as affected by neuropathic pain are characterized by higher levels of choline and phosphocholine, citrate, alanine and taurine, when compared with those of patients with nociceptive pain.

Analyzing the overall results summarized in Table 2, it is clear that a good level of classification can be obtained by pairwise OPLS-DA of ^1^H NMR spectral data of urine samples. R^2^Y values were approximately 0.80 with lower value (0.74) in the “NP vs. NC” model, thus assessing a high classification power of models. Q^2^Y values, that express the classification power in cross-validation were between 0.66 and 0.41, obviously lower than the corresponding R^2^Y. The “NP *vs*. NC” model had the lowest Q^2^Y value and a difference between R^2^Y and Q^2^Y > 0.3, indicating a weak classification performance. Interpretation of these latter results is that NP and NC can be well discriminated from controls, while when compared each other they produced less robust classifications. In general, contingency tables for all models showed high values of accuracy (between 0.79 and 0.92, Table 2 and S2 Table) all highly significant, with lower value of accuracy for “NC *vs*. C” model. In this approach we have been extremely conservative, deeming as false attributions even uncertain ones (e.g. in the “NP *vs* NC” model, the 2 false negative NP were not classified as NC, but as borderline). It is worth noting that using 0.5 as a threshold instead of 0.65 in the interpretation of OPLS-DA prediction values of belonging to a class, in order to avoid uncertain attributions (as often performed in previous literature [23]), would have led to even higher accuracy.

## Discussion

Aim of this study was to investigate whether a ^1^H NMR metabolomics approach can help in formulate a definite pain diagnosis, as it is well known that obtaining unambiguous results from clinical scores is a major issue for patients suffering from chronic pain. Very often, subjective perception of pain is influenced by other problems, such as psychological, adaptive, and conflicting statuses. Moreover, differential diagnosis between neuropathic and nociceptive pain can be very difficult, especially for non-specialists. This can lead to misdiagnosis and inappropriate treatment, exposing patients to prolonged suffering and unjustified risk of drugs adverse reactions, and leading to increasing costs for the national health systems. Our discriminating models are quite robust and the sensitivity and specificity of classification are satisfactorily high. We were able to classify subjects suffering from pain from a control group, and to differentiate neuropathic from nociceptive pain.

In general, contingency tables for all models showed high values of accuracy all highly significant. Analyzing the overall results, we can stated that NP can be better discriminated from controls than NC, indeed, lower value of accuracy was obtained when nociceptive pain and control group were compared. Although its classification power was affected by a high error (low Q^2^Y value), the model “NP *vs*. NC” showed a sensibility (accuracy = 0.86) higher than common used scores in diagnosing neuropathic pain. In fact, the efforts to identify a unique pattern of signs and symptoms for each kind of pain reached, at their best, a diagnostic certainty in about 80% of cases [1]. Indeed, not even the use of different questionnaires for the identification of pain brings fulfilling results in clinical practice. It is worth to remind that we choose to be extremely conservative, a less severe approach would have led to even higher accuracy.

As for the discriminant metabolites, we want to focus on choline and phosphocholine, alanine and taurine. The role of choline in the body is complex being involved in cell-membrane signaling (phospholipids), lipid transport (lipoproteins), methyl group metabolism (homocysteine reduction), and synthesis of the neurotransmitter acetylcholine [24]. It is the commonest polar head group of phospholipids and sphingomyelins, the major amphipathic constituents of cells membranes, the latter especially in the myelin sheath that electrically insulates the nerve cell axons. Increased levels of choline and phosphocholine are regarded as biomarkers of cellular membranes turnover in neoplasms, demyelination and gliosis [25]. As mentioned in the introduction, several authors, investigating on the metabolic basis of chronic pain, found modifications of the levels of those metabolites, among which phosphatidylcholine, involved in the sphingomyelin-ceramide metabolism [12, 17, 26]. The up-regulation of and phosphocholine found in our NP group, when compared to NC, corroborates the previous finding of a damage of nervous system linked to neuropathic pain. In fact, by its most recent definition, neuropathic pain is always associated with a nervous system lesion [27]. Alanine has been indicated as brain biomarker of apoptosis and cellular stress [28, 29]. Higher levels of alanine have been detected also in meningioma, following ischemia [1, 30, 31], and in animals encephalopathies [32]. Release of the inhibitory aminoacid taurine has been observed in neural cell damage/stress [33]. All these findings indicate that the neuropathic pain biomarkers here found, reflect neuronal damage involvement.

The lower classification power related to NC pain, either versus controls or versus NP, may be correlated to the fact that most (10 out of 12) of our NC patients suffer of osteoarticular (OA) pain. OA pain compared to NP pain, represents a still normal physiological response to an inflammatory syndrome, while NP pain generates a more extreme pathophysiological condition that can massively affects the pain pathways. Moreover, we also have to take into consideration the possible existence, in some patients with long lasting osteoarthritis, of a neuropathic component and related metabolic features, as recently postulated by Thakur et al. [34]. In this latter paper, it has been reported that the possible cause for this is the pathological spread of new neural terminations in affected joints. Therefore, the possible existence of such cases, can affect the results of our classification models, and further investigations are needed.

## Conclusions

In this exploratory study, for the first time, we investigated the ability of a ^1^H NMR metabolomics approach to differentiate between two types of chronic pain. In fact, we identified pain suffering patients from controls, and we were also able to discriminate between patients affected by neuropathic pain from those affected by nociceptive pain. The major limitation of this study is the quite low sample size, especially for the nociceptive pain group, which prevents us to generalize the obtained results. Moreover, it was hard to obtain a perfect age-matching among the various groups of subjects, due to epidemiological characteristics of the studied populations, as controls were mostly patients undergoing elective minor surgery.

As a suggestion for future development, we “throw the gauntlet” of creating a network for the best possible definition of a ^1^H NMR metabolite profile associated with different kinds of pain. Thanks to the fast growing area of technologies, nowadays a good level NMR spectrometer is relatively easy to find in Health facilities. The high reproducibility of NMR data among different laboratories may allow the collection of a huge data bank. We are not even excluding the possibility that such an approach could lead to the metabolic characterization of more than the two profiles investigated in the present work. This could be extremely important for physicians and, whenever translated into clinical practice, could allow a more accurate differential pain diagnosis, even if performed by non-specialists. If so, patients would avoid misdiagnosis and inappropriate treatment with a lowered burden of suffering and unjustified risk of drugs adverse reactions. National Health Systems would benefit from cost reductions.

## Supporting Information

S1 Fig Representative ^1^H NMR spectrum of a urine sample. Main resonances assignment is reported.
(TIFF)

S2 Fig PCA score plot of all samples. Samples are colored by A) diagnosis, B) age, and C) gender. NP: neuropathic pain, NC: nociceptive pain, C: controls.
(DOCX)

S1 Table Details of OPLS-DA models. (DOCX)

S2 Table Contingency table results. a) Pain *vs*. C, b) NP *vs*. C, c) NC *vs*. C, and d) NP *vs*. NC OPLS-DA models.
(DOCX)

S3 Table ^1^H NMR data matrix. ^1^H NMR binned spectral data. NP, neuropathic; NC, nociceptive; C, all controls; C-NP, controls matched with NP; C-NC, controls matched with NC.
(XLSX)
