# Supplementary figures and images for: Can Urine Metabolomics Be Helpful in Differentiating Neuropathic and Nociceptive Pain? A Proof-of-Concept Study
Source: PLoS One. 2016 Mar 2;11(3):e0150476. doi: 10.1371/journal.pone.0150476 (PMC4775074; doi:10.1371/journal.pone.0150476)

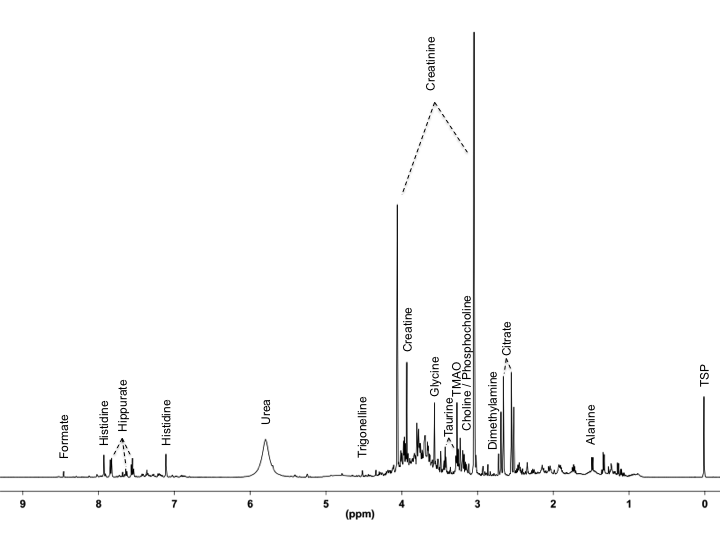

Supplement: S1 Fig — Main resonances assignment is reported. (TIFF) [file pone.0150476.s001.tiff]
