# Supplementary material for: Can Urine Metabolomics Be Helpful in Differentiating Neuropathic and Nociceptive Pain? A Proof-of-Concept Study
Source: PLoS One. 2016 Mar 2;11(3):e0150476. doi: 10.1371/journal.pone.0150476 (PMC4775074; doi:10.1371/journal.pone.0150476)
Supplement: S2 Fig — Samples are colored by A) diagnosis, B) age, and C) gender. NP: neuropathic pain, NC: nociceptive pain, C: controls. (DOCX) [file pone.0150476.s002.docx]

**S2 Fig**. **PCA score plot of all samples**. Samples are colored by A) diagnosis, B) age, and C) gender. NP: neuropathic pain, NC: nociceptive pain, C: controls,
