# Supplementary material for: Can Urine Metabolomics Be Helpful in Differentiating Neuropathic and Nociceptive Pain? A Proof-of-Concept Study
Source: PLoS One. 2016 Mar 2;11(3):e0150476. doi: 10.1371/journal.pone.0150476 (PMC4775074; doi:10.1371/journal.pone.0150476)
Supplement: S1 Table — (DOCX) [file pone.0150476.s003.docx]

**S1 Table. Details of OPLS-DA models.**

|  | **Number of samples** | | | **OPLS-DA parameters** | | | | |
| --- | --- | --- | --- | --- | --- | --- | --- | --- |
| **Model** | **NP** | **NC** | **C** | **R^2^Y** | **Q^2^Y** | **Number of validate components**  **obtained by the corresponding PLS-DA** | **Number of CV-groups^a^** | **Permutation test^b^**  **(n=400),**  **Q^2^ y-intercepts** |
| **Pain vs. C** | **25** | **12** | **37** | **0.80** | **0.66** | **2** | **5** | **-0.42** |
| **NP vs. C** | **25** | **-** | **25** | **0.83** | **0.65** | **2** | **7** | **-0.46** |
| **NC vs. C** | **-** | **12** | **12** | **0.88** | **0.64** | **2** | **6** | **-0.52** |
| **NP vs. NC** | **25** | **12** | **-** | **0.74** | **0.41** | **2** | **7** | **-0.41** |

^a^CV is performed by dividing the data into a number of groups (n=7 default) and then developing a number of parallel models from reduced data with one group deleted and used as a test set.

^b^For the permutation test, the model significance was assessed considering that R^2^ and Q^2^ for the model are larger than all the results for permuted models, and the y-intercept of the Q^2^ fit is negative.
