# Supplementary material for: Can Urine Metabolomics Be Helpful in Differentiating Neuropathic and Nociceptive Pain? A Proof-of-Concept Study
Source: PLoS One. 2016 Mar 2;11(3):e0150476. doi: 10.1371/journal.pone.0150476 (PMC4775074; doi:10.1371/journal.pone.0150476)
Supplement: S2 Table — a) Pain vs. C, b) NP vs. C, c) NC vs. C, and d) NP vs. NC OPLS-DA models. (DOCX) [file pone.0150476.s004.docx]

**S2 Table. Contingency table results.** a) Pain *vs.* C, b) NP *vs.* C, c) NC *vs*. C, and d) NP *vs*. NC OPLS-DA models

| 1. Pain *vs.* C | Pain | Control | Total |
| --- | --- | --- | --- |
| OPLS-DA Positive | 32 | 2 | 34 |
| OPLS-DA Negative | 5 | 35 | 40 |
| Total | 37 | 37 | 74 |
| Sensitivity (95% confidence interval): 0.86 (0.71 to 0.95) | | | |
| Specificity (95% confidence interval): 0.85 (0.82 to 0.99) | | | |
| Positive Predictive Value (95% confidence interval): 0.94 (0.80 to 0.99) | | | |
| Negative Predictive Value (95% confidence interval): 0.88 (0.73 to 0.96) | | | |
| P <0.0001 | | | |

| 1. NP *vs.* C | Neuropathic Pain | Control | Total |
| --- | --- | --- | --- |
| OPLS-DA Positive | 23 | 2 | 25 |
| OPLS-DA Negative | 2 | 23 | 25 |
| Total | 25 | 25 | 50 |
| Sensitivity (95% confidence interval): 0.92 (0.74 to 0.99) | | | |
| Specificity (95% confidence interval): 0.92 (0.74 to 0.99) | | | |
| Positive Predictive Value (95% confidence interval): 0.92 (0.74 to 0.99) | | | |
| Negative Predictive Value (95% confidence interval): 0.92 (0.74 to 0.99) | | | |
| P <0.0001 | | | |

| 1. NC *vs.* C | Nociceptive Pain | Control | Total |
| --- | --- | --- | --- |
| OPLS-DA Positive | 8 | 1 | 9 |
| OPLS-DA Negative | 4 | 11 | 15 |
| Total | 12 | 12 | 24 |
| Sensitivity (95% confidence interval): 0.67 (0.35 to 0.90) | | | |
| Specificity (95% confidence interval): 0.92 (0.62 to 1.00) | | | |
| Positive Predictive Value (95% confidence interval): 0.89 (0.52 to 1.00) | | | |
| Negative Predictive Value (95% confidence interval): 0.73 (0.45 to 0.92) | | | |
| P =0.01 | | | |

| 1. NP *vs.* NC | Neuropathic Pain | Nociceptive Pain | Total |
| --- | --- | --- | --- |
| OPLS-DA Positive | 22 | 2 | 24 |
| OPLS-DA Negative | 3 | 10 | 13 |
| Total | 25 | 12 | 37 |
| Sensitivity (95% confidence interval): 0.88 (0.69 to 0.97) | | | |
| Specificity (95% confidence interval): 0.83 (0.52 to 0.98) | | | |
| Positive Predictive Value (95% confidence interval): 0.92 (0.73 to 0.99) | | | |
| Negative Predictive Value (95% confidence interval): 0.77 (0.46 to 0.95) | | | |
| P <0.0001 | | | |
